# Prostaglandin F2α requires activation of calcium-dependent signalling to trigger inflammation in human myometrium

**DOI:** 10.3389/fendo.2023.1150125

**Published:** 2023-07-19

**Authors:** Lucia Riaposova, Sung Hye Kim, Aylin C. Hanyaloglu, Lynne Sykes, David A. MacIntyre, Phillip R. Bennett, Vasso Terzidou

**Affiliations:** ^1^ Parturition Research Group, Institute of Reproductive and Developmental Biology, Department of Metabolism, Digestion and Reproduction, Imperial College London, London, United Kingdom; ^2^ The March of Dimes European Prematurity Research Centre at Imperial College London, London, United Kingdom; ^3^ The Parasol Foundation Centre for Women’s Health and Cancer Research, St Mary’s Hospital, Imperial College Healthcare National Health Service (NHS) Trust, London, United Kingdom; ^4^ Department of Obstetrics & Gynaecology, Chelsea and Westminster Hospital National Health Service (NHS) Trust, London, United Kingdom

**Keywords:** prostaglandin F2 alpha (PGF2α), myometrium, cyclooxygenase-2 (COX-2), inflammation, preterm birth, labour, G protein-coupled receptor (GPCR)

## Abstract

**Introduction:**

Preterm birth is one of the major causes of neonatal morbidity and mortality across the world. Both term and preterm labour are preceded by inflammatory activation in uterine tissues. This includes increased leukocyte infiltration, and subsequent increase in chemokine and cytokine levels, activation of pro-inflammatory transcription factors as NF-κB and increased prostaglandin synthesis. Prostaglandin F2α (PGF2α) is one of the myometrial activators and stimulators.

**Methods:**

Here we investigated the role of PGF2α in pro-inflammatory signalling pathways in human myometrial cells isolated from term non-labouring uterine tissue. Primary myometrial cells were treated with G protein inhibitors, calcium chelators and/or PGF2α. Nuclear extracts were analysed by TranSignal cAMP/Calcium Protein/DNA Array. Whole cell protein lysates were analysed by Western blotting. mRNA levels of target genes were analysed by RT-PCR.

**Results:**

The results show that PGF2α increases inflammation in myometrial cells through increased activation of NF-κB and MAP kinases and increased expression of COX-2. PGF2α was found to activate several calcium/cAMP-dependent transcription factors, such as CREB and C/EBP-β. mRNA levels of NF-κB-regulated cytokines and chemokines were also elevated with PGF2α stimulation. We have shown that the increase in PGF2α-mediated COX-2 expression in myometrial cells requires coupling of the FP receptor to both Gαq and Gαi proteins. Additionally, PGF2α-induced calcium response was also mediated through Gαq and Gαi coupling.

**Discussion:**

In summary, our findings suggest that PGF2α-induced inflammation in myometrial cells involves activation of several transcription factors – NF-κB, MAP kinases, CREB and C/EBP-β. Our results indicate that the FP receptor signals via Gαq and Gαi coupling in myometrium. This work provides insight into PGF2α pro-inflammatory signalling in term myometrium prior to the onset of labour and suggests that PGF2α signalling pathways could be a potential target for management of preterm labour.

## Introduction

Preterm birth (PTB) is defined by the World Health Organisation as birth completed before the 37th week of gestation (1). Every year, approximately 15 million babies are born preterm, with rates of preterm birth increasing worldwide (2). Preterm birth and the associated complications are the major cause of neonatal death and illness around the world. Preterm labour shares many common pathways with term labour, however, preterm labour results from early activation of these pathways (3). Towards the end of pregnancy and in labour, the uterus goes through a set of gradual biochemical, anatomical, endocrine and immune changes that transform it from a quiescent state maintaining pregnancy, to the activated contractile state, ready for labour (4–6). Key transformations within reproductive tissues include cervical remodelling and dilatation, fetal and decidual membrane activation and an increase in myometrial contractility (5, 7–9). An increase in myometrial contractility results from changes in expression of proteins that promote intercellular connectivity and excitability of myocytes (6, 10–12). Through the increasing activity of metalloproteinases in the cervix, the structure of the cervix changes and begins to soften and dilate (13–16). Activation of decidua and fetal membranes involves the degradation of extracellular matrix proteins and consequential weakening of the membranes (9, 17). This leads to their separation from decidua in the lower uterine segment and eventually their rupture (17, 18).

Although the full mechanism that controls and triggers the onset of labour is yet to be understood, several studies have shown that the onset of both term and preterm labour is associated with the activation of inflammatory pathways (19–21). Parturition is characterised by an increased leukocyte infiltration into the uterus with the levels of cytokines and chemokines such as IL-6, IL-8, CCL2, CCL5, TNF-α and IL-1β being elevated in term and preterm labour (22–25). Several cytokines (including IL-1β and TNF-α) contribute to an increase in prostaglandin production by activating phospholipid metabolism and stimulating cyclooxygenase 2 (COX-2) expression (26). IL-6 and IL-1β increase oxytocin secretion with IL-6 also increasing the expression of the oxytocin (OT) receptor (27, 28). Cooperative effects of IL-1β and prostaglandin F2α (PGF2α) on IL-6 and COX-2 upregulation in myometrial cells were recently described (29). IL-1β, IL-6 and TNF-α stimulate translocation of nuclear factor-κB (NF-κB) to the nucleus and thus triggering the transcription of several pro-inflammatory mediators (30). In preterm labour, the stretch and infiltrating pro-inflammatory cytokines can further increase the expression of chemokines in myometrium through activation of NF-κB (24). Additionally, the upregulation of a cassette of genes related to uterine activation and myometrial contractility takes place in the myometrium prior to labour (31). These so-called, contraction-associated proteins (CAPs), include ion channel proteins, connexin-43 and the receptors for oxytocin and prostaglandins.

The prostaglandin biosynthetic pathway is activated prior to the onset of labour with cytosolic phospholipase A_2_ (PLA_2_) and COX-2 enzymes participating in key steps of the prostaglandin biosynthesis in reproductive tissues (32, 33). In the uterus, prostaglandin E2 (PGE2) and PGF2α stimulate myometrial contractility (4), and contribute to parturition-associated fetal membrane activation and rupture through stimulation of matrix metalloproteinase activity, leading to extracellular matrix degradation (32, 34). Additionally, prostaglandin-mediated matrix metalloproteinase (MMP) activity contributes to cervical ripening (35–37). PGF2α activates signalling pathways that lead to the activation of NF-κB, mitogen-activated protein kinases (MAPK), phosphatidylinositol-4,5-bisphospate 3-kinase (PI3K) and calcineurin/nuclear factor of activated T-cells (NFAT) in myometrial cells (38). Additionally, it has been reported that PGF2α also increases the expression of several uterine activation proteins (such as connexin-43, oxytocin receptor (OTR), COX-2) (39) and pro-inflammatory cytokines and chemokines in myometrium through the activation of phospholipase C (PLC) and protein kinase C (PKC) pathways (40). In turn, these cytokines drive further production of prostaglandins resulting in a feed-forward loop.

The effects of PGF2α are mediated through its FP receptor, belonging to a family of G protein-coupled receptors (GPCRs). The FP receptor signals through interactions with multiple G proteins, including Gαq/11, Gα12/13 and Gβγ (presumed to originate from Gαi) (41–44). In human myometrium, the coupling of the FP receptor to Gαq/11 has been shown to activate PLCβ (42) leading to the release of intracellular calcium essential for myometrial contraction, however, the role of G proteins in PGF2α-mediated inflammation is yet to be elucidated. This study aims to investigate the signalling pathways involved in pro-inflammatory signalling mediated by PGF2α in human myometrium with the focus on G protein-mediated signalling, transcription factor activation and the role of calcium.

As PGF2α drives myometrial contractility via FP receptor coupling to a Gαq/11-calcium pathway, we hypothesised that PGF2α-mediated inflammation in human myometrium is via coupling to Gαi. This could highlight a role for specific FP receptor/G protein signalling pathways to be exploited as potential therapeutic target for PTB prevention. By profiling the effects of PGF2α on inflammatory pathway transcription factors, MAP kinases, cytokines and COX-2, we investigated the involvement of Gαi and Gαq coupling and calcium in PGF2α-mediated signalling in primary myometrial cells.

## Methods

### Myometrial tissue collection

Myometrial tissue was collected from patients undergoing planned caesarean sections at term prior to labour onset. Myometrial tissue was taken from the upper margin of the lower uterine segment incision and kept in phosphate-buffered saline (PBS) at 4°C until further processing. Myometrial tissue samples were obtained with informed written consent. This research study was approved by the local ethics committees (Riverside Ethics Committee (Ref 3358), and London Harrow Research Ethics Committee (Ref 19/LO/1657).

### Primary myometrial smooth muscle cell culture

For isolation of myometrial smooth muscle cells, myometrial tissue was washed in phosphate-buffered saline and dissected into small pieces within 24 hours of collection. Dissected tissue was then digested in the filter-sterilised (0.2μm) collagenase solution (collagenase 1A 1mg/ml, collagenase 1mg/ml, bovine serum albumin 2mg/ml dissolved in a 50:50 mixture of serum-free high glucose formulation of Dulbecco’s Modified Eagle’s Medium (DMEM) and DMEM/Nutrient F-12 HAM) for 45 minutes at 37°C. The enzymatic activity was stopped by addition of complete DMEM containing 10% fetal bovine serum (FBS), 2mM L-glutamine and 100 U/mL penicillin-streptomycin. The mixture was filtered through a cell strainer (40μm) and cells were isolated by centrifugation at 3000 rpm (1841xg) for 2 minutes. Cells were resuspended in complete DMEM and seeded into a culture flask (T25) to be grown at 37°C, 5% CO_2_. Cells were subcultured when they reached 90-95% confluence. Cells were washed with PBS and incubated in Trypsin-EDTA solution (0.5 g/L porcine trypsin and 0.2 g/L EDTA x 4Na in Hanks′ Balanced Salt Solution with phenol red) for 5 minutes. The enzyme activity was stopped by addition of DMEM containing 10% FBS. The cell suspension was centrifuged at 1000 rpm (205xg) for 5 minutes. The cells were resuspended in complete DMEM and seeded into flasks or plates. Primary myometrial smooth muscle cells were used for all the experiments presented in this study. Cells were used between passage numbers 2 and 6.

To investigate PGF2α-mediated inflammatory signalling in myometrium, human myometrial cells were treated with PGF2α (1µM). The selected concentration was previously reported to stimulate myometrial contractility and to increase the uterine activation proteins expression (39, 40, 45).

### Whole cell protein extraction

For whole cell protein extraction, modified radioimmunoprecipitation assay (RIPA) buffer (1% (v/v) Triton X-100, 1% (w/v) Sodium deoxycholate, 0.1% (v/v) SDS, 150mM NaCl, 10mM Tris, pH 7.4, 1mM EDTA, 1mM PMSF, 0.5% v/v protease inhibitor cocktail, 1% v/v phosphatase inhibitor cocktail) was used. Cell monolayers were scraped off with a cell scraper and collected in 1.5ml microfuge tubes. Cell lysates were centrifuged at 13300xg for 30 minutes at 4°C. Supernatants were transferred to clean microfuge tubes and stored at -20°C until further analysis.

### Nuclear/cytosolic protein extraction

Nuclear and cytosolic proteins were extracted from fresh cells. Following completion of treatment, the culture media were removed from the wells and 60 µl of cytosolic extraction buffer A was added to each well. Cells were scraped with plate scraper and the lysate was left to incubate on ice for 10 minutes. 20% Nonidet P-40 was added to each tube (to a final concentration of 1%) and the tubes were vortexed for 10 seconds. The lysates were then centrifuged at 13300xg for 30 seconds at 4°C. The supernatants containing the cytosolic fraction were transferred to clean microfuge tubes, snap-frozen on dry ice and stored at -80°C. The pellet was resuspended in 40µl of nuclear buffer B and the lysates were incubated on ice while shaking for 15 minutes. The lysates were centrifuged at 13300xg for 5 minutes at 4°C. The supernatants containing the nuclear protein fraction were transferred to clean microfuge tubes, snap-frozen on dry ice and then stored at -80°C.

### Protein quantification

Protein concentration of whole cell extracts were determined by using detergent compatible (DC) protein assay reagents (Bio-Rad) following the manufacturer’s protocol. Briefly, 20µl of reagent S was added to each ml of reagent A. 25µl of the working mixture was added to 5 µl of standards and samples in 96-well microtiter plates. Then, 200 µl of reagent B was added to the wells. Samples and standards were prepared in duplicates. After 15 minutes of incubation, absorbance was read at 650nm using Optimax microplate reader. Bovine serum albumin standards of known concentrations which were prepared in protein extraction buffer were used as a reference to determine the protein concentration of the lysates.

### Western blotting

Extracted protein samples were denatured with protein loading buffer at 80°C for 10 minutes. Protein samples (10-20μg) were loaded onto precast Tris-Glycine polyacrylamide gels (4-20%, Bio-Rad) and resolved by electrophoresis (100V for first 10 minutes, followed by 140V for 60-90 minutes). Following electrophoresis, proteins were transferred to a 0.2µm polyvinylidene fluoride membrane by a semi-dry transfer using Bio-Rad TransBlot Turbo Blotting System (2.5A (constant), 7 minutes). Membranes were blocked in 5% skimmed milk solution in Tris-buffered saline with Tween (TBS-T) (w/v) for 1 hour and then were hybridised with primary antibody at 4°C overnight. Following the incubation with primary antibodies, membranes were washed in TBS-T and then incubated with secondary antibodies conjugated to horseradish peroxidase (HRP) for 1h at room temperature. Membranes were washed in TBS-T and the target proteins were detected using Clarity Western ECL Substrate (Bio-Rad) or Pierce ECL 2 Western Blotting Substrate (Thermo Scientific). ImageQuant LAS4000 Luminescent Image Analyser (GE Healthcare) and Amersham Hyperfilm ECL (GE Healthcare) were used to image the bands. Densitometric analysis was performed using ImageQuant TL v8.1 (GE Healthcare).

### RNA extraction

Total RNA was extracted by guanidium thiocyanate-phenol-chloroform extraction using RNA STAT-60 (Amsbio) following the manufacturer’s recommendation. The cells in each well were lysed with 600μl of RNA STAT-60. Cell monolayer was scraped with a clean filter tip, the lysate was passed through a pipette tip several times and transferred to a clean microfuge tube. The homogenate was kept at room temperature for 5 minutes. Chloroform (Sigma-Aldrich) (0.2ml per 1ml of RNA STAT-60) was added to each microtube, the tubes were vortexed for 15 seconds and then centrifuged at 12000xg for 15 minutes at 4°C. The aqueous phase was transferred to microtubes containing 0.5ml of isopropanol. The microtubes were left at room temperature for 15 minutes and then centrifuged at 13300xg for 20 minutes at 4°C. The supernatant was removed, and the pellets were washed with 0.5ml of 75% ethanol and then centrifuged at 12000xg for 10 minutes. The supernatant was discarded, and the pellets were allowed to air dry. RNA was resuspended in 20μl of DEPC-treated water. The concentration and the purity of the extracted RNA were determined using NanoDrop ND-1000 spectrometer. The RNA extracts were stored at -80°C.

### DNase treatment and first-strand cDNA synthesis

DNase I was used to remove any potential contamination with DNA in the RNA extracts. 2μg of RNA was mixed with DEPC-treated water, 1μl of DNase I (Sigma-Aldrich) and 1μl of Reaction Buffer (Sigma Aldrich) in the total reaction volume of 20μl. The reaction mix was incubated at room temperature for 15 minutes. The reaction was inactivated by the addition of 1μl of Stop Solution and subsequent heating of the mixture for 10 minutes at 70°C. The reaction mixture was chilled on ice.

DNase I-treated reaction mix was incubated with 1μl 10mM Deoxynucleotide Mix (Sigma-Aldrich) and 1μl Oligo-dT (Sigma-Aldrich) for 10 minutes at 70°C and then chilled on ice. 10μl M-MLV Reverse Transcriptase Buffer, 1μl M-MLV Reverse Transcriptase, 0.5μl Ribonuclease Inhibitor and 6.5μl DEPC-treated water were added to the mixture and the mixture was incubated at room temperature for 15 minutes, then at 37°C for 50 minutes, and then at 85°C for 10 minutes. cDNA was stored at -20°C until further analysis.

### Real-time PCR

Quantification of target cDNA was performed by real time polymerase chain reaction with SYBR^®^ Green JumpStart™ Taq ReadyMix™ (Sigma-Aldrich) and the primers specific for the target cDNA (**Table 1**). Real time PCR was carried out in 96-well plates using StepOnePlus Real Time PCR system (Applied Biosystems). Each reaction mix (total volume: 20μl) contained 10μl SYBR^®^ Green JumpStart™ Taq ReadyMix™, 0.2μl Reference Dye for Quantitative PCR, 0.3μl forward primer (20nM), 0.3μl reverse primer (20nM), 5.2μl DEPC-treated water, and 4μl of diluted cDNA. Reactions were run in duplicates. Following cycling conditions were used: 2 min at 95°C, 40 to 45 cycles of 15 sec at 95°C, 30 sec at 60°C, 100 sec at 72°C; melt curve: 15 sec at 85°C, 1 min at 60°C and 15 sec at 95°C. Gene expression levels (cycle threshold values) for all target genes were normalised to the expression levels of the constitutively expressed gene GAPDH. The comparative cycle threshold method (2^-ΔΔCt^) was used to determine relative differences in gene expression.

**Table 1:** Sequences of primers used in real-time PCR.

Gene	Forward Sequence	Reverse Sequence
GAPDH	TGATGACATCAAGAAGGTGGTGAAG	TCCTTGGAGGCCATGTAGGCCAT
IL-6	CCTTCCAAAGATGGCTGAAA	AGCTCTGGCTTGTTCCTCAC
IL-8	GCCTTCCTGATTTCTGCAGC	CGCAGTGTGGTCCACTCTCA
IL-1β	GCTGAGGAAGATGCTGGTTC	TCCATATCCTGTCCCTGGAG
CCL2	TCTGTGCCTGCTGCTCATAG	AGATCTCCTTGG CCACAATG
CCL5	CCATATTCCTCGGACACCAC	TGTACTCCCGAACCCATTTC
COX-2	GCTCAAACATGATGTTTGCATTC	GCTGGCCCTCGCTTATGA

### Intracellular calcium response

Myometrial cells were seeded in individual 35mm dishes with 14mm glass microwells (MaTek Corporation) and were grown to near confluence in DMEM – high glucose medium containing 10% FBS, 2mM L-glutamine and 100 U/ml penicillin-streptomycin. Prior to treatment, the cells were kept in serum-depleted growth medium (DMEM - low glucose with/containing 1% FBS, 2mM L-glutamine and 100 U/ml penicillin-streptomycin) overnight. An equal volume of 2X Fluo-4 Direct™ calcium reagent loading solution was added to culture dishes containing cells with growth medium. The cells were incubated with the calcium reagent for 30 minutes at 37°C and then for 30 minutes in the dark at room temperature. Fluorescence intensity was measured using Leica SP5 confocal microscope (Objective: HC PL FLUOTAR 20.0x0.50 DRY) with the settings for excitation at 494 nm and emission at 516 nm. Basal level of fluorescence was measured for 3 minutes and the fluorescence intensity levels in response to treatments were measured for 15 minutes. Fluorescence intensity values in response to treatments were normalised to basal level of fluorescence recorded for non-stimulated cells. Fluorescent intensity plots and the maximum fluorescence intensity values were recorded using Leica Application Suite Advanced Fluorescence Lite Software and analysed using MS Excel and GraphPad Prism. Fluorescence intensity values are expressed in arbitrary units (AU).

### Transcription factor array

TranSignal cAMP/Calcium Protein/DNA Array (Panomics) was used to identify transcription factors that become activated with PGF2α receptor stimulation (**Table 2**). Myometrial cells were treated with PGF2α (1µM, 30 minutes) and nuclear extracts were used for the protein/DNA array protocol as per manufacturer’s protocol.

**Table 2:** Transcription factors screened in TranSignal protein/DNA array.

AP-1	Activator protein 1
C/EBP	CCAAT-enhancer-binding proteins
CBF	CCAAT-binding factor (CCAT- enhancer binding protein zeta)
CREB	cAMP response element-binding protein
E4F, ATF	E4F transcription factor, Activating transcription factor family
EGR	Early growth response protein
Ets	Erythroblast transformation specific transcription factor family
GATA-3	GATA binding protein 3 (Trans-acting T-cell-specific transcription factor GATA-3)
GATA-4	GATA binding protein 4
HNF-4	Hepatocyte nuclear factor 4
HSE	Heat shock transcription factor
MEF-2	Myocyte-specific enhancer factor 2A
NFAT	Nuclear factor of activated T-cells
NF-E1	Yin-Yang-1 transcription factor
NF-κB	nuclear factor kappa-light-chain-enhancer of activated B cells
OCT-1	Octamer-binding transcription factor 1 (POU domain, class 2, transcription factor 1)
PPARγ	Peroxisome proliferator-activated receptor gamma
Rel	Proto-oncogene c-Rel
Smad SBE	Mothers against decapentaplegic homolog, SMAD binding elements
Sp1	Specificity protein 1

Nuclear extracts (3-5μg/μl) from PGF2α-treated myometrial cells were incubated with TranSignal Probe Mix (biotin-labelled DNA binding oligonucleotides) to allow the formation of DNA/protein complexes. The protein/DNA complexes (TF-bound probes) were isolated from free probes by spin column separation. The labelled probes from the DNA/protein complexes were then extracted and hybridised to TranSignal array membrane.

For detection, the membranes were blocked in a blocking buffer (Panomics), incubated with streptavidin-HRP conjugate (Panomics) and then washed in wash buffer (Panomics). Membranes were then incubated with detection buffer (Panomics) and enhanced chemiluminescence reagents (Panomics). Membranes were exposed using chemiluminescence imaging system - ImageQuant LAS4000 luminescent image analyser (GE Healthcare) and Amersham Hyperfilm ECL (GE Healthcare). The density of the spots was measured using Fiji ImageJ and ImageQuant TL v8.1 (GE Healthcare). The local area surrounding each spot was used for background subtraction. The density values of each spot were normalised to the average density of the positive control spots on each membrane. Ratio between treated and non-stimulated controls was calculated. Two-fold increase in spot density was considered significant (as per manufacturer’s recommendation).

### Statistical analysis

Statistical analysis was performed on all presented data. Data was tested for normality using Kolmogorov- Smirnov test. Normally distributed data was analysed by analysis of variance (ANOVA) followed by Tukey’s or Dunnett’s multiple comparisons tests. Data that was not normally distributed was analysed using Kruskal-Wallis test with Dunn’s multiple comparisons test. All analyses were carried out using GraphPad Prism 9. All results are presented as means of biological replicates with standard errors of the mean (SEM), and probability value of p<0.05 was considered statistically significant.

## Results

### PGF2α increases inflammation in human myometrium

PGF2α is a uterotonic agent that also participates in uterine activation prior to labour (39). To study the effects of PGF2α on inflammatory signalling pathways involving NF-κB, MAP kinases and COX-2 in myometrium, cultured human myometrial cells were treated with PGF2α (1µM) and the levels of target proteins were assessed by Western blotting.

**Figure 1:**
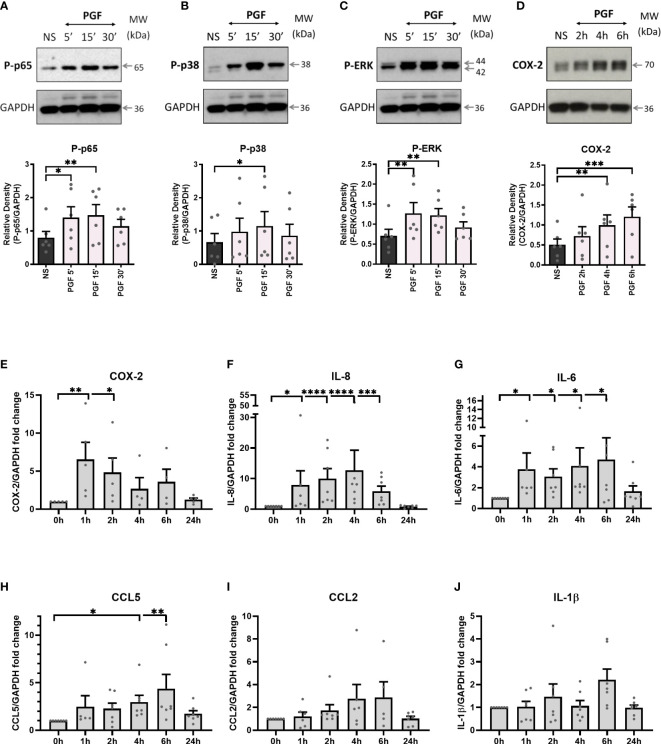
Prostaglandin F2α increases inflammation in myometrium. Primary myometrial smooth muscle cells isolated from term non-labouring myometrium were stimulated with prostaglandin F2α (1μM) for 5 min, 15 min, 30 min, 1h, 2h, 4h, 6h, and 24h. Proteins levels of phospho-p65 **(A)**, phospho-p38 **(B)**, phospho-ERK **(C)** and COX-2 **(D)** following treatment with PGF2α were analysed by Western blotting. Graphs are showing relative densities of proteins normalised to GAPDH. Individual repeats are presented as dots. Representative blots (including the loading control) are shown above their corresponding densitometry graphs (n=6) (NS – non-stimulated, PGF - prostaglandin F2α). Total RNA was extracted from myometrial cells that were treated with PGF2α. mRNA levels of COX-2 **(E)**, IL-8 **(F)**, IL-6 **(G)**, CCL5 **(H)**, CCL2 **(I)** and IL-1β **(J)** were measured using RT-qPCR. Results in the graphs are expressed as fold change relative to non-stimulated control. (n=5-7); * p < 0.05, ** p<0.01, *** p<0.001, **** p<0.0001 vs NS, ANOVA with Dunnett’s post-test).

Treatment of term myometrial cells with PGF2α resulted in the activation of p65 subunit of NF-κB (p<0.01 at 15 min), MAP kinases p38 (p<0.05 at 15 min) and Extracellular signal-regulated kinase (ERK) (p<0.01 at 15 min) (**Figures 1A–C**). Additionally, PGF2α increased the expression of COX-2 (p<0.001 at 6h), a rate-limiting enzyme in prostaglandin synthesis (**Figure 1D**). It has been well established that NF-κB participates in the regulation of pro-inflammatory gene expression associated with the onset of labour (46). Therefore, RT-qPCR was used to examine the effects of PGF2α stimulation on downstream NF-κB-regulated gene expression. As expected, the levels of COX-2 mRNA increased significantly within 1h of PGF2α treatment resulting in a 6-fold increase in expression (p<0.01) (**Figure 1E**). Moreover, PGF2α upregulated multiple pro-labour cytokines and chemokines including IL-8, IL-6 and CCL5 by 12.7-fold (p<0.0001 at 4h), 4.7-fold (p<0.05 at 6h), and 4.4-fold (p<0.01 at 6h), respectively (**Figures 1F–H**). The expression of CCL2 (2.9-fold) and IL-1β (2.2-fold) mRNA showed increasing trend with PGF2α stimulation but did not reach statistical significance (**Figures 1I, J**). The effects of PGF2α on downstream NF-κB regulated genes were transient with the mRNA levels returning to basal levels by 24h.

### The effects of PGF2α on COX-2 require both Gαq and Gαi coupling

**Figure 2:**
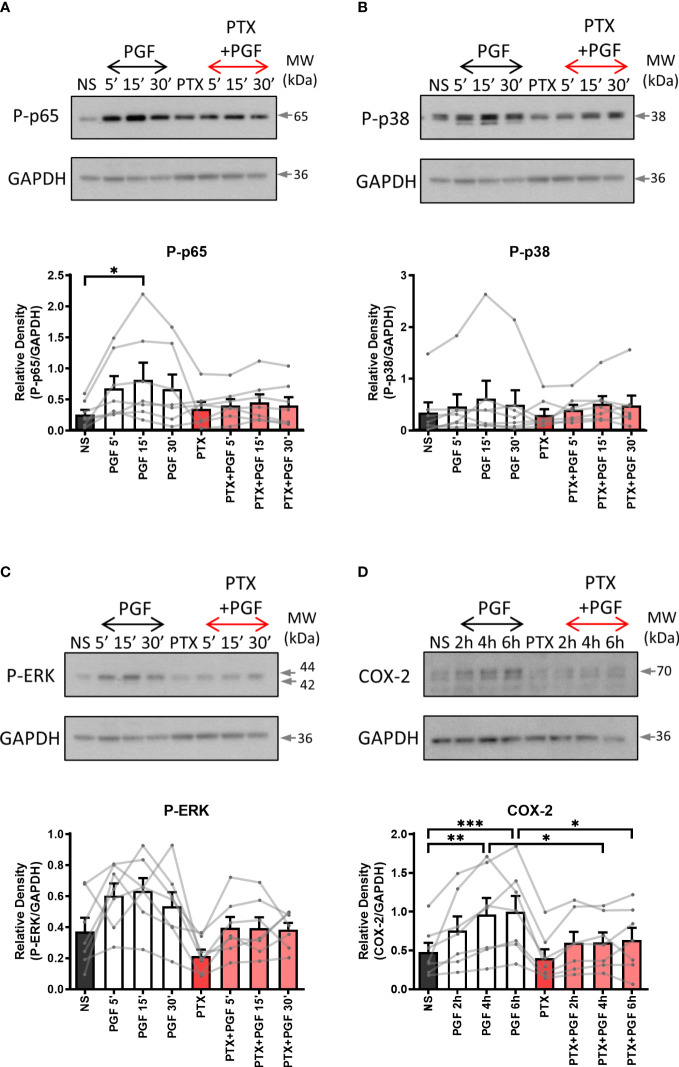
The inhibition of Gαi coupling reduces prostaglandin F2α-mediated COX-2 expression. Primary myometrial cells isolated from term non-labouring myometrium were pre-treated with pertussis toxin (PTX, 200ng/ml) prior to stimulation with PGF2α (1μM). Changes in the protein levels of phospho-p65 **(A)**, phospho-p38 **(B)**, phospho-ERK **(C)** and COX-2 **(D)** were measured using Western blotting. Individual repeats are presented as dots connected with lines. Representative Western blots are shown above their corresponding densitometry graphs. Values are presented as mean ± SEM (n=7, ANOVA with Tukey post-test, * p < 0.05, ** p<0.01, *** p<0.001 vs NS or PGF2α-treated).

**Figure 3:**
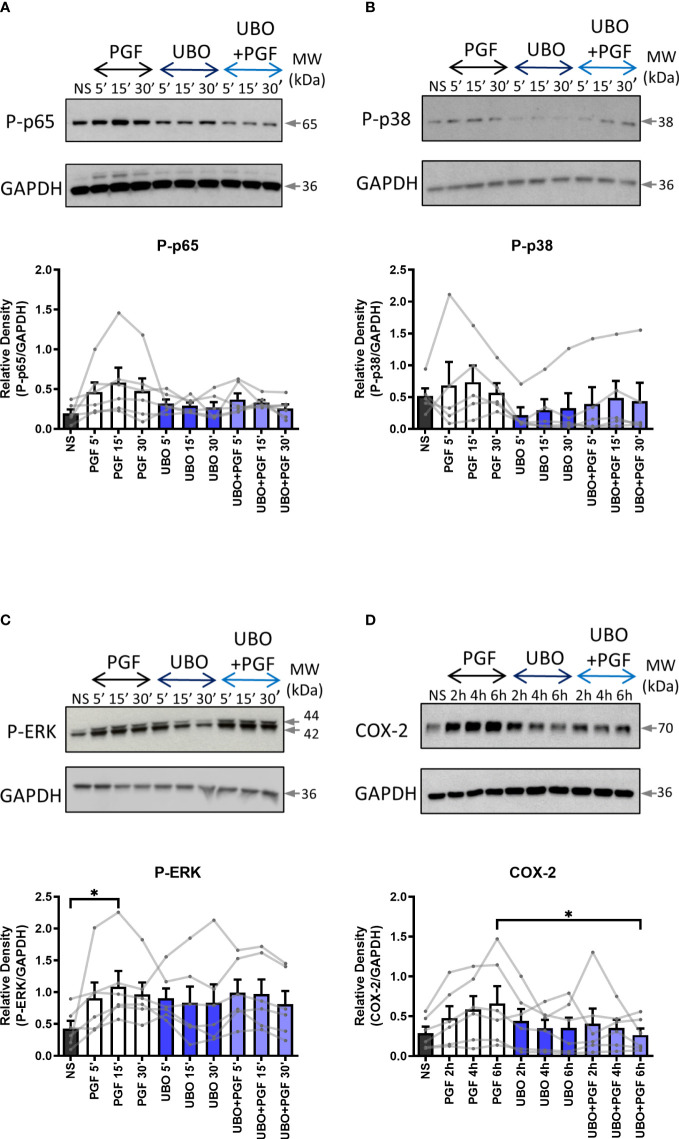
The inhibition of Gαq coupling reduces prostaglandin F2α-mediated COX-2 expression. Primary myometrial cells isolated from term non-labouring myometrium were pre-treated with UBO-QIC (1μM) prior to stimulation with PGF2α (1μM). Changes in the protein levels of phospho-p65 **(A)**, phospho-p38 **(B)**, phospho-ERK **(C)** and COX-2 **(D)** were measured using Western blotting. Individual repeats are presented as dots connected with lines. Representative Western blots are shown above their corresponding densitometry graphs. Values are presented as mean ± SEM (n=5-6, ANOVA with Tukey post-test, * p<0.05 vs NS or PGF2α-treated).

As the FP receptor can couple to multiple G proteins, we aimed to determine the role of Gαq and Gαi proteins in PGF2α-stimulated pro-inflammatory signalling. Myometrial cells were pre-treated either with Gαi protein inhibitor (pertussis toxin, PTX, 200 ng/ml) (**Figures 2A-D**) or with Gαq inhibitor (UBO-QIC (1μM)) (**Figures 3A-D**) and then stimulated with PGF2a (1μM). The concentrations of G protein inhibitors were selected based on previously published studies (47–50). Treatment of myometrial cells with PTX decreased the PGF2α-stimulated activation of ERK MAP kinase (38% decrease at 15 min) and significantly reduced the upregulation of COX-2 (p<0.05 at 6h) (**Figures 2C, D**) but did not have a clear effect on NF-κB p65 or p38 MAP kinase activation (**Figures 2A, B**). Notably, treatment with the Gαq inhibitor, UBO-QIC, also resulted in reduced PGF2α-induced expression of COX-2 (p<0.05 at 6h), potentially via NF-κB activation which was decreased by 43% at 15 min (**Figures 3A, D**). These results show that PGF2α-driven COX-2 upregulation requires both Gαq and Gαi coupling. We suggest that FP receptor coupling to Gαi could potentially regulate COX-2 expression via activation of ERK, whereas the mechanism through which Gαq regulates COX-2 may involve NF-κB but this requires further investigation.

### PGF2α-mediated calcium response is driven through both Gαq and Gαi protein coupling

**Figure 4:**
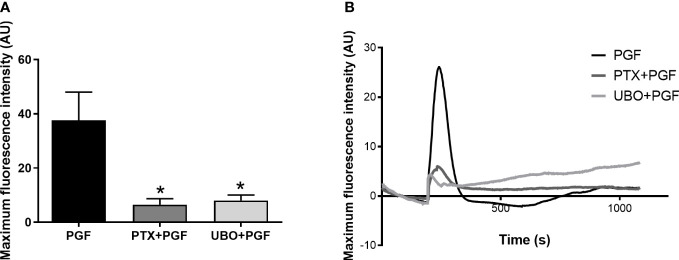
Gαi and Gαq coupling are involved in calcium response to prostaglandin F2α stimulation in myometrial cells. Primary myometrial cells isolated from term non-labouring myometrium were loaded with calcium sensitive dye, Fluo-4-Direct, and stimulated with PGF2α in the presence or absence of G protein inhibitors: PTX or UBO-QIC. Maximum fluorescence intensities **(A)** are expressed as mean ± SEM in arbitrary units (AU) (n=4, Kruskal-Wallis test with Dunn’s post-test). * p<0.05 vs NS or PGF2α-treated. Average plots **(B)** for mean fluorescent intensity for each treatment expressed in AU.

FP receptor activation and G protein coupling increases intracellular calcium concentration which can result from an influx of calcium ions across plasma membrane or through calcium release from internal stores (51). We have shown that the FP receptor couples to both Gαq and Gαi in response to PGF2α binding. Through pre-treatment with G protein inhibitors, we confirmed that both G proteins are involved in the calcium response following PGF2α stimulation as calcium response was significantly reduced when Gαq (p<0.05) and Gαi (p<0.05) proteins were inhibited in myometrial cells (**Figures 4A, B**).

### PGF2α-mediated calcium response plays a role in NF-κB activation and upregulation of COX-2

**Figure 5:**
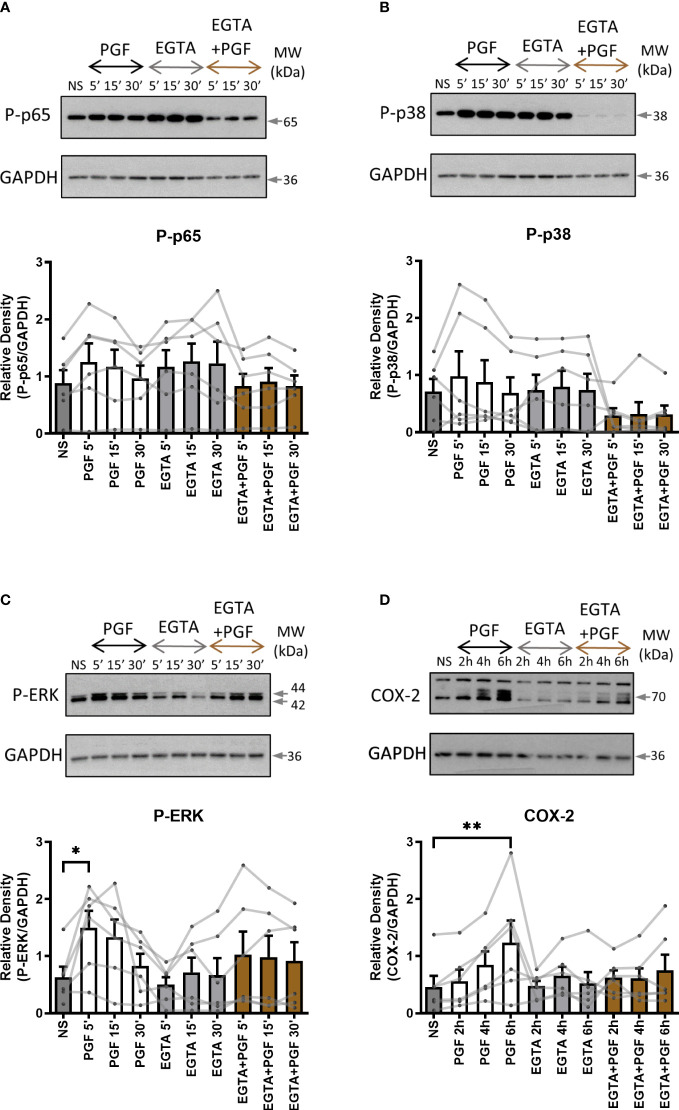
The role of extracellular calcium in prostaglandin F2α-mediated pro-inflammatory signalling. Primary myometrial smooth muscle cells isolated from term non-labouring myometrium were pre-treated with EGTA (1 mM) prior to stimulation with PGF2α. Changes in the protein levels of phospho-p65 **(A)**, phospho-p38 **(B)**, phospho-ERK **(C)** and COX-2 **(D)** were measured using Western blotting. Individual repeats are presented as dots connected with lines. Representative Western blots are shown above their corresponding graphs. Values are presented as mean ± SEM (n=6, * p < 0.05, ** p<0.01 vs NS, ANOVA with Tukey post-test).

**Figure 6:**
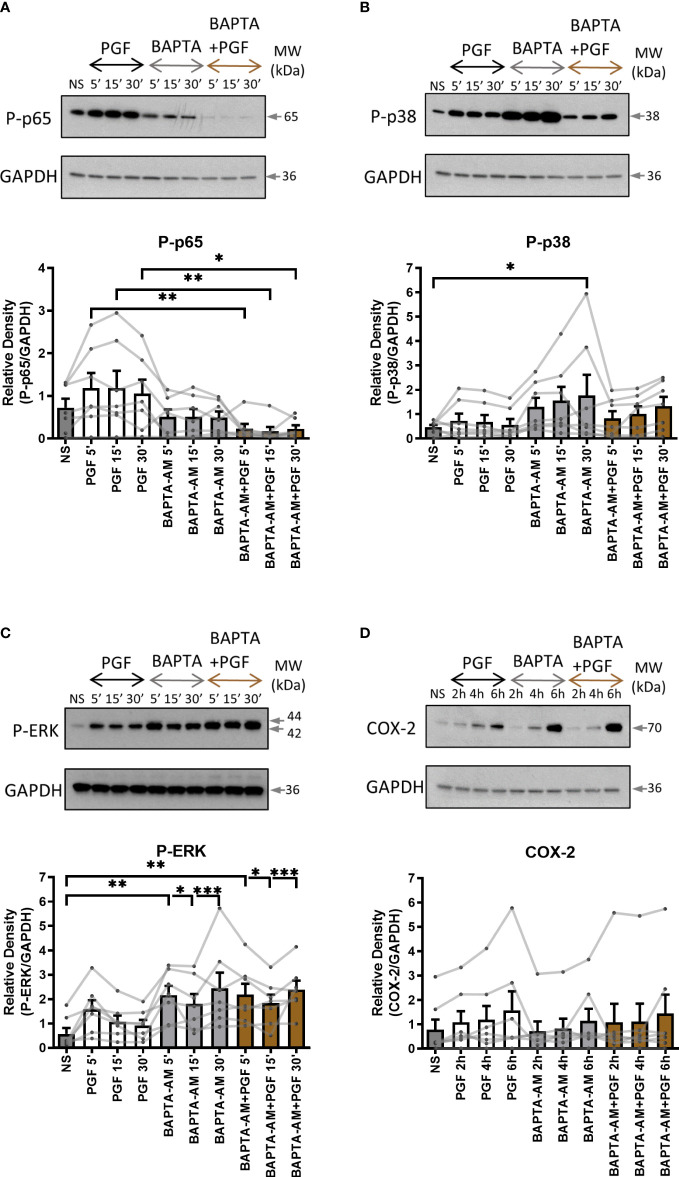
The chelation of extracellular and intracellular calcium decreases prostaglandin F2α-mediated activation of NF-κB. Primary myometrial smooth muscle cells isolated from term non-labouring myometrium were pre-treated with BAPTA-AM (10μM) prior to stimulation with PGF2α. Changes in the protein levels of phospho-p65 **(A)**, phospho-p38 **(B)**, phospho-ERK **(C)** and COX-2 **(D)** were measured using Western blotting. Individual repeats are presented as dots connected with lines. Representative Western blots are shown above their corresponding graphs. Values are presented as mean ± SEM (n=7, ANOVA with Tukey post-test, * p < 0.05, ** p<0.01, *** p<0.001 vs NS or PGF2α-treated).

To examine the role of extracellular and intracellular calcium in pro-inflammatory signalling in myometrium, primary myometrial cells were pre-treated with extracellular calcium chelator EGTA (1mM) for 2 hours or intracellular chelator BAPTA-AM (10µM) for 30 minutes prior to stimulation with PGF2α (1µM). The concentration of calcium chelators was selected based on concentrations used in previously published studies (47, 52, 53). Depleting extra- or intra- cellular calcium with calcium chelators had distinctively different effects on PGF2α-mediated activation of NF-κB, MAP kinases and COX-2 expression in myometrial cells. There was a decreasing trend in p38 MAP kinase activation (70% decrease at 5 min) and a decrease in COX-2 expression (39% decrease at 6h) with the chelation of extracellular calcium (**Figures 5B, D**) but little effect on p65 and ERK activation (**Figures 5A, C**). The depletion of intracellular calcium with BAPTA-AM treatment significantly reduced the activation of p65 subunit of NF-κB (p<0.01 at 15 min) (**Figure 6A**), however, BAPTA-AM by itself appeared to induce MAP kinase activation (phospho-p38: p<0.05 at 30 min; phospho-ERK: p<0.001 at 30 min) and COX-2 expression (1.5-fold), therefore, it was not possible to determine the effects of intracellular calcium depletion on these proteins (**Figures 6B–D**). These results indicate that calcium originating from both outside the cell and intracellular stores may play a role in PGF2α-induced pro-inflammatory signalling.

### PGF2α activates a range of transcription factors in myometrial cells

**Figure 7:**
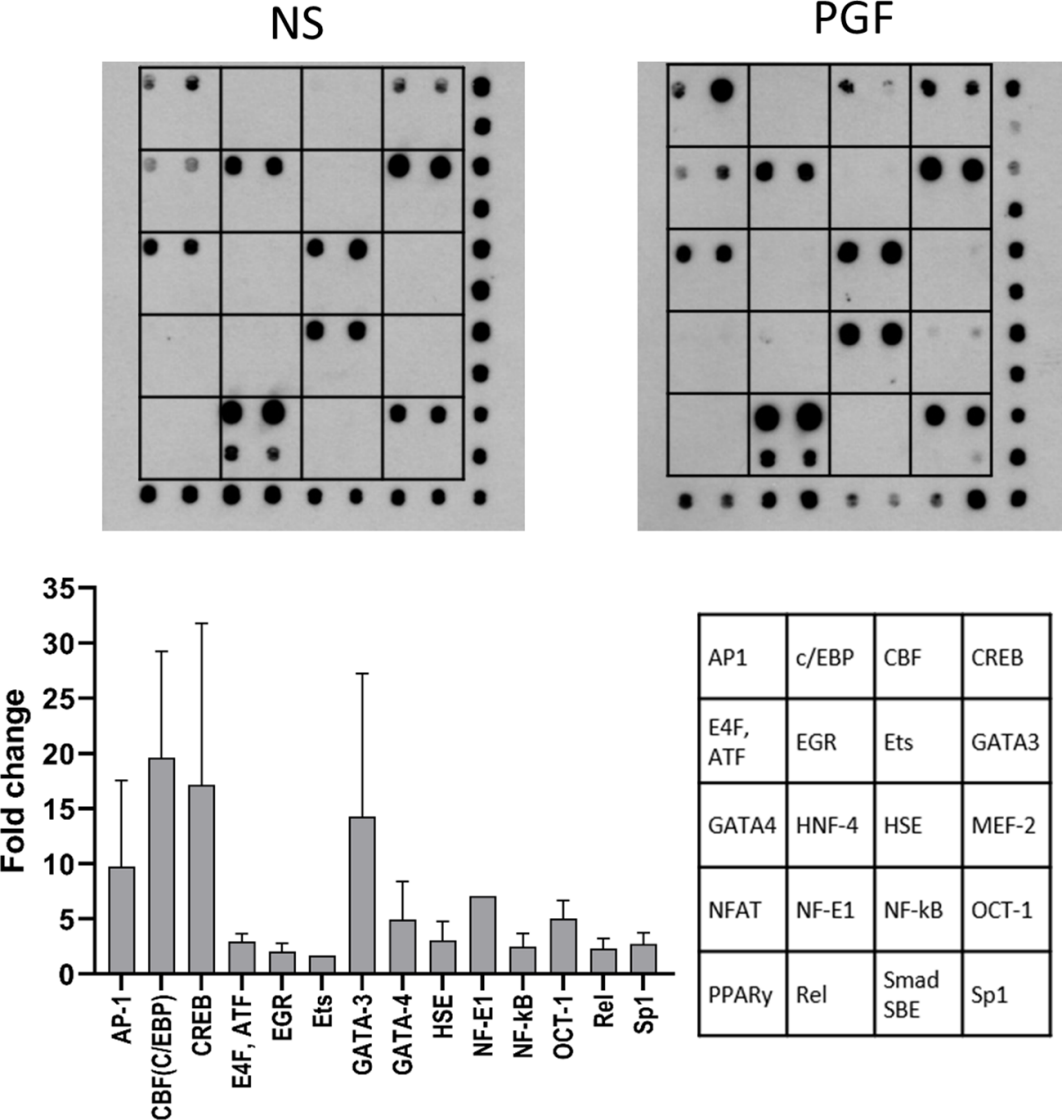
Prostaglandin F2α increases the activation of cAMP/calcium-regulated transcription factors. Primary myometrial cells isolated from term non-labouring myometrium were treated with PGF2α (1µM, 30 minutes). The activity of cAMP/calcium regulated transcription factors was determined using TranSignal cAMP/Calcium Protein/DNA Array. The graph shows the fold change increase for the transcription factors whose activity increased more than 1.5-fold. Values are presented as mean ± Range (n=2). Representative array dot blots for non-stimulated and PGF2α-treated samples are shown above. (AP-1 - Activator protein 1, C/EBP - CCAAT-enhancer-binding proteins, CBF - CCAAT-binding factor (CCAT- enhancer binding protein zeta), CREB - cAMP response element-binding protein, E4F, ATF - E4F transcription factor, Activating transcription factor family, EGR - Early growth response protein, Ets - Erythroblast transformation specific transcription factor family, GATA-3 - GATA binding protein 3 (Trans-acting T-cell-specific transcription factor GATA-3), GATA-4 - GATA binding protein 4, HNF-4 - Hepatocyte nuclear factor 4, HSE - Heat shock transcription factor, MEF-2 - Myocyte-specific enhancer factor 2A, NFAT - Nuclear factor of activated T-cells, NF-E1 - Yin-Yang-1 transcription factor, NF-κB - nuclear factor kappa-light-chain-enhancer of activated B cells, OCT-1 - Octamer-binding transcription factor 1 (POU domain, class 2, transcription factor 1), PPARγ - Peroxisome proliferator-activated receptor gamma, Rel - Proto-oncogene c-Rel, Smad SBE - Mothers against decapentaplegic homolog, SMAD binding elements, Sp1 - Specificity protein 1).

We have shown that PGF2α couples to Gαq and Gαi to regulate COX-2 expression. We have demonstrated that PGF2α activates NF-κB and MAP kinases. However, a wide range of transcription factors can be activated by G-protein and calcium signalling and these may be regulating COX-2 expression. Therefore, to identify the detailed mechanism involved in PGF2α-induced inflammation, protein/DNA array (TranSignal™ cAMP/Calcium Protein/DNA Array) was used to examine the effects of PGF2α on additional transcription factors associated with calcium response (n=2). Treatment of myometrial cells with PGF2α increased the activation of several calcium/cAMP-regulated transcription factors. Twenty transcription factors were tested and 14 were found to be upregulated by more than 2-fold (**Figure 7**). The stimulation of myometrial cells with PGF2α markedly increased the activation of AP1 (9.8-fold), C/EBP (19.6-fold), CREB (17.1-fold), GATA-3 (14.2-fold), GATA-4 (4.9-fold), NF-E1 (7.1-fold), and OCT-1 (5-fold). The following transcription factors were also upregulated by more than 2-fold with PGF2α treatment but to a smaller extent than the transcription factors above: ATF (2.9-fold), EGR (2.1-fold), HSE (3.1-fold), NF-κB (2.5-fold), Rel (2.3-fold) and Sp-1 (2.7-fold).

### PGF2α activates calcium-regulated transcription factors via G protein coupling

**Figure 8:**
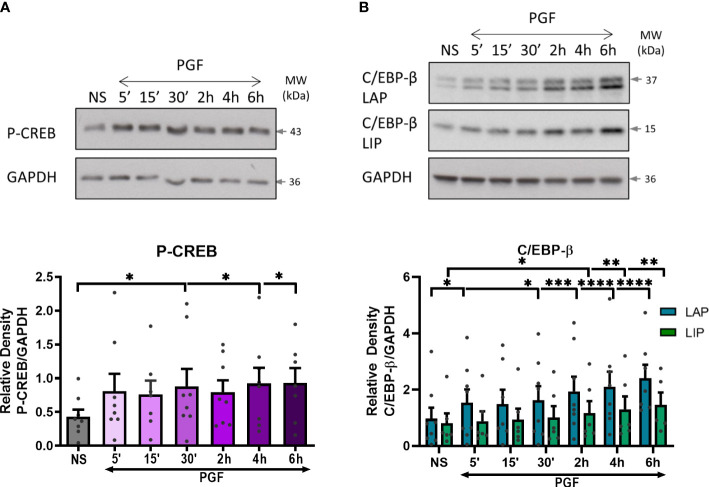
Prostaglandin F2α increases CREB and C/EBP-β activation in myometrium. Primary myometrial cells isolated from term non-labouring myometrium were treated with PGF2α (1μM) for 5, 15, 30 minutes and 2, 4, 6 hours. Changes in the protein levels of phospho-CREB **(A)** and C/EBP-β **(B)** were measured using Western blotting. Individual repeats are presented as dots. Representative Western blots (including the loading control, GAPDH) are shown above their corresponding densitometry graphs. Values are presented as mean ± SEM (n=5, ANOVA with Dunnett’s post-test; * p<0.05, ** p<0.01, *** p<0.001, **** p<0.0001 vs NS).

From the protein-DNA array, we have seen a high level of upregulation in CREB and C/EBP following PGF2α stimulation. As the COX-2 promoter region contains binding sites for CREB and C/EBP-β transcription factors, we investigated the effects of PGF2α on cAMP/calcium-regulated transcription factors CREB and C/EBP-β. The results showed increased levels of phospho-CREB as early as 5 minutes after PGF2α treatment and remained elevated for the duration of the treatment (p<0.05 at 30 min) (**Figure 8A**). The levels of the activating C/EBP-β LAP subunit increased following 5 minutes (p<0.05) of treatment and increased further during longer treatment time periods (2.5-fold increase, p<0.0001 at 6h), whereas the levels of the inhibitory C/EBP-β LIP subunit were increased to a lesser extent than C/EBP-β LAP (1.6-fold increase, p<0.01 at 6h) (**Figure 8B**) resulting in an increase in C/EBP-β LAP to LIP ratio over time.

**Figure 9:**
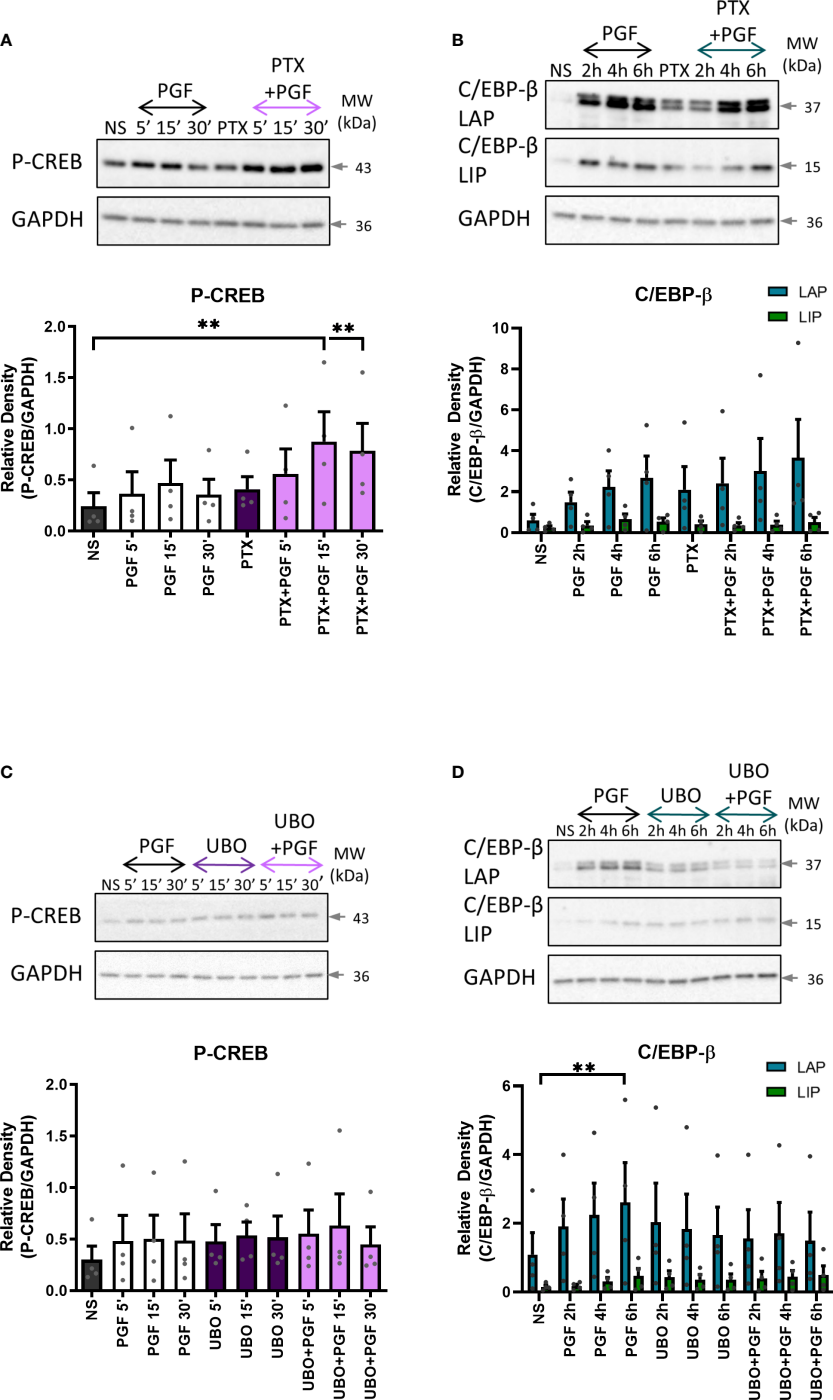
The role of Gαi and Gαq protein coupling in prostaglandin F2α-mediated activation of cAMP/calcium-dependent transcription factors. Primary myometrial cells isolated from term non-labouring myometrium were pre-treated with pertussis toxin (200ng/ml) **(A, B)** or UBO-QIC (1μM) **(C, D)** prior to stimulation with PGF2α (1μM). Changes in the protein levels of phospho-CREB **(A, C)** and C/EBP-β **(B, D)** were measured using Western blotting. Individual repeats are presented as dots. Representative Western blots are shown above their corresponding densitometry graphs. Values are presented as mean ± SEM (n=4, ANOVA with Tukey post-test, ** p<0.01 vs NS).

To determine the role of Gαq and Gαi proteins in PGF2α-mediated activation of CREB and C/EBP-β, myometrial cells were pre-treated with either Gαi or Gαq inhibitor. There was no decrease in CREB or C/EBP-β activation with Gαi inhibition (**Figures 9A, B**). However, there was a decreasing trend in the activation of C/EBP-β LAP with Gαq inhibition (43% decrease at 6h) but no effect on CREB activation (**Figures 9C, D**), suggesting that PGF2α-mediated activation of C/EBP-β could possibly occur via Gαq signalling pathways.

## Discussion

**Figure 10:**
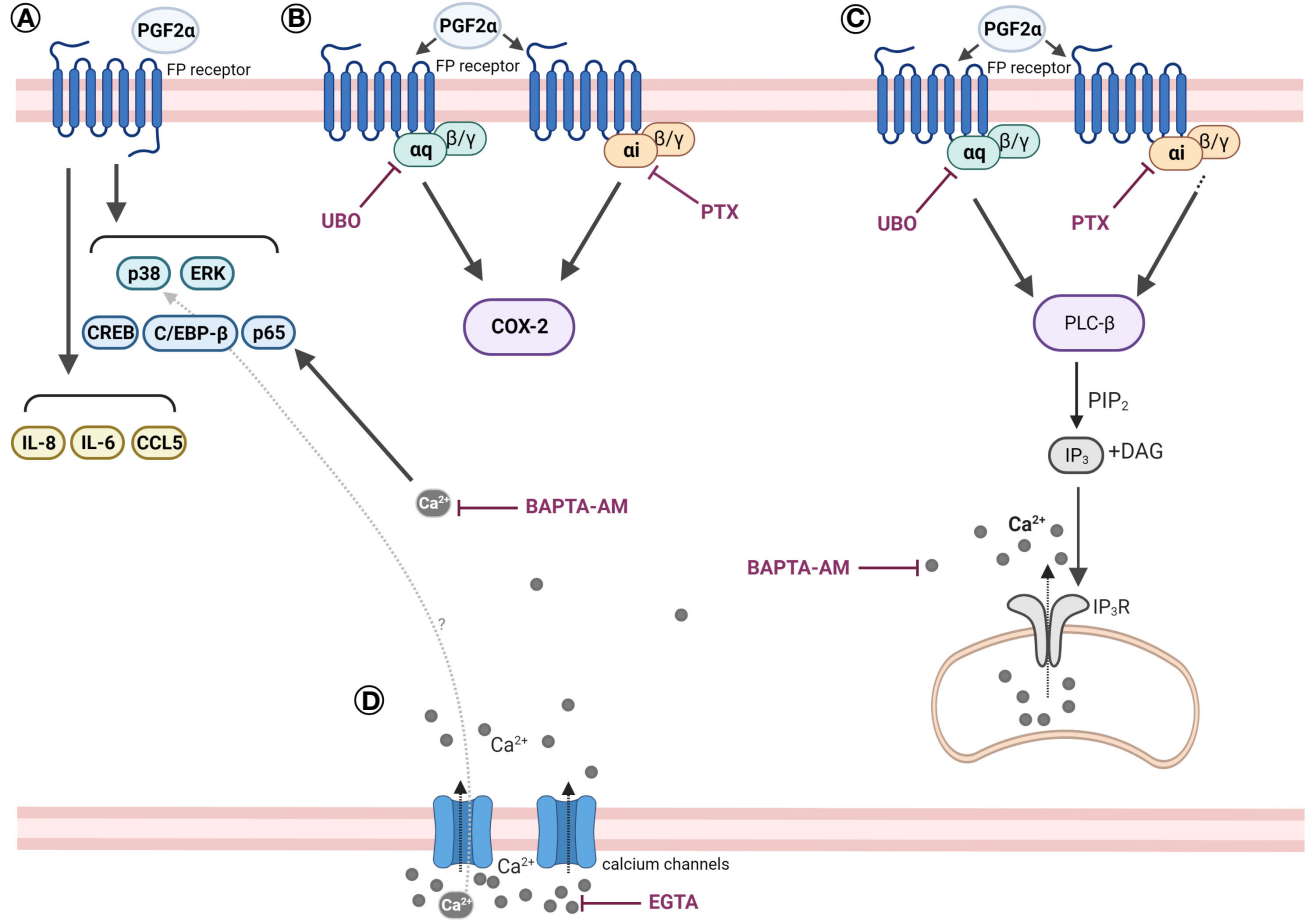
Scheme summarising the effects of PGF2α in human myometrial smooth muscle cells. **(A)** PGF2α induces pro-inflammatory signalling in myometrial cells. PGF2α increases the activation of NF-κB, MAP kinases, CREB, and C/EBP-β. PGF2α upregulates the expression of IL-8, IL-6, CCL5. **(B)** PGF2α-mediated COX-2 upregulation requires both Gαi and Gαq protein coupling of the FP receptor. **(C)** PGF2α stimulation triggers a calcium response in myometrial cells via Gαq and Gαi-mediated pathways. **(D)** PGF2α-mediated calcium response plays a role in NF-κB activation. Extracellular calcium influx might play a role in PGF2α-mediated p38 MAP kinase activation. Created with BioRender.com. BAPTA-AM, 1,2-bis(o-Aminophenoxy)ethane-N,N,N’,N’-tetraacetic acid tetrakis(acetoxymethyl ester) (calcium chelator); CCL5, Chemokine (C-C motif) ligand 5; C/EBP-β, CCAAT/enhancer-binding protein beta; COX-2, Cyclooxygenase 2; CREB, cAMP response element-binding protein; DAG, Diacylglycerol; EGTA, Ethylene glycol-bis(β-aminoethyl ether)-N,N,N’,N’-tetraacetic acid (calcium chelator); ERK, Extracellular signal-regulated kinase; IL-6, Interleukin 6; IL-8, Interleukin 8; IP3, Inositol trisphosphate; IP3R, Inositol trisphosphate receptor; PGF2α, Prostaglandin F2α; PIP2, Phosphatidylinositol 4,5-bisphosphate; PLC-β, Phospholipase C beta; PTX, Pertussis toxin; p38, p38 mitogen-activated protein kinase; UBO, UBO-QIC (FR900359).

It is widely accepted that term and preterm labour are preceded by inflammatory activation within the uterine tissues. Building on our current understanding of PGF2α as an inflammatory mediator, we have demonstrated that PGF2α activates the calcium response and pro-inflammatory signalling pathways in human myometrium through Gαq and also Gαi coupling of the FP receptor (**Figure 10**). PGF2α increases the activation of NF-κB, MAP kinases, CREB and C/EBP-β, and in turn upregulates the expression of cytokines and chemokines as well as COX-2. PGF2α-mediated COX-2 upregulation has been shown to require both Gαi and Gαq protein coupling of the FP receptor. Additionally, we have shown that the calcium response to PGF2α stimulation in myometrial cells occurs via Gαq and Gαi-mediated pathways.

PGF2α is a potent uterotonin, however, the role of PGF2α goes beyond the stimulation of myometrial contractions. PGF2α plays an important signalling role during uterine transformation prior to labour onset. We found that in myometrial cells, PGF2α increased the activation of p65 subunit of NF-κB, which is a prototypical pro-inflammatory transcription factor. It regulates the expression of several pro-inflammatory and pro-labour genes and thus plays an important role in the onset of term and preterm labour (54–56). Additionally, PGF2α increases transcription and protein levels of the inducible COX-2 enzyme. This indicates that there is a positive feed-forward loop between PGF2α and COX-2 in myometrium which is complementary to the findings of Xu et al. (39). Growing evidence suggests that parturition is characterised by infiltration of leukocytes into the uterus leading to increased expression and release of pro-inflammatory cytokines and chemokines (23, 57, 58). PGF2α can contribute to the establishment of a pro-labour environment in the uterus through promoting an increase in expression of pro-inflammatory and chemotactic cytokines such IL-8, IL-6, CCL5, IL-1β and CCL2. The increased activation of NF-κB and MAP kinases in response to PGF2α stimulation might be one of the driving forces for the increased cytokine output by myometrial cells. Several genes encoding pro-inflammatory cytokines and chemokines contain the NF-κB binding site in their promoters (55, 59). NF-κB activation upregulates genes for CCL2, CCL5, IL-8 and IL-6 (56). Moreover, it has been reported that IL-1β, IL-6 and CCL2 production by myocytes is ERK-dependent, with the production of IL-1β and CCL2 also being NF-κB-dependent (40). Increased levels of chemotactic cytokines in the uterus promote the recruitment of more leukocytes to further increase additional cytokine production (60–62), resulting in a positive feed-forward loop (40, 63). Pro-inflammatory cytokines IL-1β and IL-6, which are released by invading leukocytes, have the ability to activate NF-κB and C/EBP, resulting in subsequent expression of inflammatory mediators and uterine activation proteins such as OT receptor and COX-2 (30, 64, 65).

Our study indicates that the activity of the PGF2α receptor in human myometrium is mediated by coupling to several G proteins. Sales et al. have previously reported that in endometrial adenocarcinoma cell line, PGF2α-mediated upregulation of COX-2 occurs via Gαq signalling pathway (66). Here we report that inhibition of Gαq and Gαi coupling reduces PGF2α-induced COX-2 expression in human myocytes, therefore indicating that both G proteins are required to mediate the effects of PGF2α on COX-2 expression. Similar to our findings, Ohmichi et al. reported that in addition to traditional Gαq coupling, PGF2α has the ability to activate MAPK through Gβγ originating from Gαi coupling in rat puerperal myometrial cells (41). Studies in rat hepatocytes and rabbit kidney cells have suggested that the FP receptor can couple to Gαi protein (67, 68).

Our previous studies are indicative of potential crosstalk between PGF2α and oxytocin (OT) receptors. We reported that oxytocin receptor antagonists atosiban and nolasiban were able to inhibit both OT-induced and PGF2α-induced myometrial contractions and a novel tocolytic, OBE002, an FP receptor antagonist, reduced PGF2α-induced and OT-induced contractions (45, 69). The FP receptor has been previously reported to form heterodimers with angiotensin II type I receptor (AT1R) in HEK293 cells and vascular smooth muscle cells with the heteromer showing marked differences in signalling in comparison to monomers (70, 71). Direct physical interaction between the FP receptor and the OT receptor has not been studied or reported before, however functional crosstalk can also occur in the absence of physical interaction (72). This receptor crosstalk could affect many aspects of PGF2α-regulated signalling pathways as the OT receptor couples to several G proteins (48, 73). OT-mediated Gαq coupling leads to increased myometrial contractility, however, it has been shown that oxytocin mediates inflammatory effects in human gestational tissues *via* Gαi activation (48). It is therefore possible that Gαi-mediated effects on COX-2 that we have observed in response to PGF2α stimulation could be a result of FP receptor and OT receptor interaction. Further studies are needed to examine the presence of FP/OT receptor heteromers and to determine the functional downstream implications of such interaction.

Uterine contractions are initiated through an increase in intracellular calcium concentrations in myometrial cells (74). It has been well established that FP receptor coupling to Gαq leads to the mobilisation of intracellular calcium (42, 75). Our findings suggest that coupling of the FP receptor to both Gαq and Gαi proteins can trigger an increase in intracellular calcium concentration in myocytes. Typically, a receptor coupling to Gαq protein family leads to PLCβ activation which converts phosphatidylinositol 4,5-bisphosphate (PIP2) into diacylglycerol (DAG) and inositol trisphosphate (IP3), leading to a stimulation of calcium release from intracellular stores. However, in addition to this traditional pathway of PLCβ activation, it has been previously reported that PLCβ isoforms can be activated by Gβγ subunit which originates from Gαi complex (76–79). As we have shown that Gαi coupling plays a role in PGF2α-induced COX-2 expression, it is possible that this alternative pathway can at least partially mediate the effects of PGF2α on PLCβ activation and subsequent calcium response. Our results indicate that the FP receptor in myometrium couples to Gαq and Gαi proteins. Further investigation of G protein coupling to a wider range of G proteins using primary myometrial cells with knocked down individual G proteins would help us complete the picture of PGF2α-mediated pro-inflammatory signalling in myometrium, as the understanding the signalling pathways involved in PGF2α-mediated effects is essential to our understanding of parturition.

PGF2α, acting through its receptor elicits a strong calcium response in myometrial cells, mainly through release of calcium from stores. Several reports suggest that PGF2α stimulation of myometrial cells leads to an influx of calcium ions from extracellular space either through voltage-gated calcium channels or capacitative/store-controlled calcium entry (51, 75). PGF2α also triggers calcium release from intracellular stores, which is mediated through PLCβ activation (42, 51, 80–83). Our results indicate that calcium participates as a second messenger in pro-inflammatory signalling pathways. PGF2α-stimulated activation of NF-κB is affected by availability of intracellular calcium whereas extracellular calcium might play a role in PGF2α-induced p38 MAPK activation and COX-2 expression. Calcium-dependent agonist-induced activation of transcription factors has been previously reported in literature in several cell types (84–89). Through a transcription factor array, we have identified that PGF2α activates 14 out of 20 calcium-regulated transcription factors (90). Of these, C/EBP-β activation by PGF2α has been shown to be via Gαq coupling which could drive COX-2 expression. Transcriptional regulation of COX-2 by C/EBP-β has been previously demonstrated in various cell types such as fibroblasts, vascular and cardiac endothelial cells (91). Overall, our results suggest that PGF2α-mediated signalling may require presence of calcium ions to regulate various aspects of the pro-inflammatory response.

This study has potential limitations. The aim of the study was to investigate PGF2α-induced pro-inflammatory signalling in myometrium in order to evaluate FP receptor-mediated signalling pathways as potential targets for preterm labour treatment and prevention. Our study investigated responses to PGF2α stimulation in cells isolated from lower uterine segment. Although, uterine regional differences in pro-labour gene expression have been reported (92), primary myometrial smooth muscle cells from upper and lower segment have the ability to respond to stimulation with IL-1β and PGF2α resulting in increases in COX-2 expression and cytokine production (39, 40, 92). We have also previously demonstrated that activation of NF-κB occurs in both upper and lower segment of the uterus (56). However, it would be beneficial to study and compare the G protein activation and calcium responses in the cells from the upper uterine segment in the future. Another limitation of our study is a wide inter-sample variability which may be confounding some of the results. The myometrial smooth muscle cells used in this study were isolated from term non-labouring uterine tissue, however some patients may have been closer to labour onset than others. Towards the end of gestation, the uterus undergoes an array of phenotypic changes that culminate in labour (93). Transcriptional and gene expression analysis revealed differences in gene expression between labouring and non-labouring myometrial tissue (19, 94). Basal level of uterine activation therefore differs between patient samples and can alter their ability to respond to stimuli (55).

PGF2α plays a major role in pathophysiology of preterm labour through its role in inflammatory activation of myometrium and via stimulation of myometrial contractility. The FP receptor therefore appears to be a good target for pharmacological management of preterm labour. However, the signalling pleiotropy associated with GPCRs presents a challenge in designing effective therapeutics. A widely used OTR antagonist, atosiban, was shown to activate pro-inflammatory pathways in amnion epithelial cells through biased agonist effect on the OT receptor (48). Moreover, a study by Fillion et al. proposed asymmetric recruitment of β-arrestins in AT1R/FP dimers (70). These studies collectively highlight the need for the development of compounds that would modify specific signalling pathways, particularly when targeting G protein-coupled receptors.

In summary, this study has demonstrated the induction of pro-inflammatory response by PGF2α in primary human myometrial cells. The results of our study suggest that upregulation of COX-2 by PGF2α may be linked to upstream activation of MAP kinase, NF-κB and C/EBP-β, and involves calcium-dependent signal transduction by Gαi and Gαq proteins. These findings contribute to our understanding of myometrial preparation for the onset of labour and highlight the need to further investigate myometrium-specific mechanisms in order to develop tocolytic drugs with better uterine selectivity.

## Data availability statement

The raw data supporting the conclusions of this article will be made available by the authors, without undue reservation.

## Ethics statement

The studies involving human participants were reviewed and approved by Riverside Ethics Committee (Ref 3358), London Harrow Research Ethics Committee (Ref 19/LO/1657). The patients/participants provided their written informed consent to participate in this study.

## Author contributions

LR, SK, AH, LS, DM, PB and VT contributed to conception and design of the study. LR performed the experiments. LR, SK and VT analysed and interpreted the data. LR and SK wrote the first draft of the manuscript and prepared the figures. All authors contributed to critical revision of the manuscript, read and approved the submitted version.
